# Editorial: Hand and Touch: Evolution, Ability, and Preference

**DOI:** 10.3389/fpsyg.2017.01930

**Published:** 2017-11-01

**Authors:** Jacqueline Fagard, Miriam Ittyerah

**Affiliations:** ^1^Université Paris Descartes, CNRS, Paris, France; ^2^Department of Psychology, University of Delhi, New Delhi, India

**Keywords:** handedness, haptics, asymmetries, development, evolution

Indices of behavioral asymmetries can be traced back to millions of years (trilobite), long before appendages appeared. Today, asymmetries can be observed at the population-level in species lacking limbs. However, many studies have shown that increasing degree of manual skill increases asymmetry in hand selection, in humans as well as non-humans. The hand has two main functions, a motor function (grasping, holding, etc.) and a haptic function (touching, exploring, etc.). In as much as asymmetries reflect brain specialization, the more skillful hand may vary according to the most solicited function. In this special issue, seven articles discuss the impact of haptic contribution, task complexity, and other factors in the expression of hand or limb preference, in infants and adult humans, and in other species.

Grasping is one of the most studied manual skills. In particular, for developmental psychologists interested in the origin of handedness, grasping is one of the first behaviors in which infant's handedness can be evaluated (the first one, thumb sucking, appears *in utero* and is related to future handedness). Based on their own and others' infant studies, Streri and de Hevia showed that the right hand is more efficient than the left hand for holding already at birth. They also described how hand asymmetry can be tested on higher perceptive functions, such as cross-modal transfer, memory, analytic vs. global processing using the habituation paradigm. Interestingly, when a newborn is presented with an object in hand in the absence of vision, s/he shows visual recognition of the shape after tactual habituation with the right but not with the left hand.

Grasping itself can be evaluated in more or less complex conditions. In most infant studies, handedness was evaluated when the infant grasps a stationary object. Grasping a moving object requires anticipation of the action. In this condition, Domellöf et al.'s study showed a change in strategy between 8 and 10 months of age. At 8 months, infants mostly used their right-hand to anticipate and grasp the moving object whatever its starting position (right, middle, or left), whereas 10-month-olds adapted their use of one hand to the starting position of the object, mostly using the ipsilateral hand. Changes in hand selection with age may be related to changes in other domains, such as capacity for planning the action or locomotor skills.

Most daily activities require both hands and bimanual handedness has been somewhat neglected as compared to unimanual handedness. Gonzalez and Nelson wrote a brief review of studies concerning role-differentiated bimanual manipulation that involves actions when the two hands assume a different and complementary role (holding and manipulating). Role-differentiated bimanual manipulation emerges during the first year of life and becomes lateralized around the end of the first year, with the majority of 18–24 months children exhibiting a right-hand preference for the active component of the action and a left-hand preference for holding the object. Gonzalez and Nelson recommend longitudinal studies including a sufficient number of trials to evaluate handedness for bimanual manipulation.

Grasping can occur without vision. Even in conditions of normal vision, knowledge of the object is not complete until the object is fully secured in hand when haptic processing adds new information. In their impressive review, Stone and Gonzalez remind us how vision and haptics combine and interact for grasping, the relative importance of each sensory channel depending on the context, the goal of the grasp, the object, etc. One interesting question is whether manual asymmetries in grasping change according to the relative importance of vision and haptics. Experimental, brain imaging, and neuropsychological studies with data from innate or acquired deficits support the notion that the left hemisphere is specialized for precise grasping under vision whereas the right hemisphere is specialized for haptic discrimination of objects by the right as well as by the left hand.

Some manual skills reveal a very stable selection of hand over time, for instance writing, which is a highly practiced skill, or hammering which requires precision. Others are less stable, such as manipulating a remote control device. Several grasping studies have been devoted to understand the main factors influencing hand selection. Scharoun et al.'s study presented in this issue extends existing literature to show that hand selection for grasping is a function of spatial compatibility, object characteristics and affordances, intention following grasping, number of actions in anticipation, and uni- vs. bimanuality of the action. Thus, biomechanical, cognitive, and spatial constraints all modulate the motor predominance of the right hand in right-handers and to a lesser extent, the left hand in left-handers.

Limb asymmetries are not exclusively human, and are observed at the population level in non-human primates and more generally across non-human vertebrates, and even invertebrates. There is a lack of consensus as to whether great apes show population-level right-handedness or whether their right-handedness is task-specific. In their article, Meguerditchian et al. confirm the impact of task characteristics (such as complexity) and individual factors (such as preferred grip or postural habits) on the degree of right-handedness. In their study of 564 great apes, they observed right-handedness at the population level in bimanual tasks more than simple grasping, in apes using a thumb-index finger grip more than in apes using other types of grips, and more in less arboreal species (bonobos, chimpanzees, and gorillas) than in orangutans.

In a comprehensive review of forelimb preference in humans and other species, Versace and Vallortigara indicate some very important points that may serve as conclusions to the afore mentioned issue: (1) it is still a matter of debate whether the degree and consistency of forelimb asymmetries distinguish human from non-human laterality; (2) forelimb preferences are particularly evident in feeding, tool-use, and bimanual coordinated actions, are enhanced by the demands of the task, and are influenced by other factors such as posture or social context, thus reflecting brain asymmetries not only for motor control but also for other lateralized functions (reaction to stress, social life, emotions); (3) although experience plays a role in developing asymmetries, population, and biochemical genetic studies point to a possible multifactorial complex genetic influence; (4) language is not necessary to show population-level limb preferences but the degree of right-handedness is generally stronger for communicative gestures than for grasping objects; (5) comparative and anthropological evidence seem to indicate very ancient and multi evolutionary origins of this trait.

In conclusion, these studies indicate how important it is to consider vision, haptics, task-related, contextual, and individual factors to understand the early emergence and evolution of hand preference.

## Author contributions

All authors listed have made a substantial, direct and intellectual contribution to the work, and approved it for publication.

### Conflict of interest statement

The authors declare that the research was conducted in the absence of any commercial or financial relationships that could be construed as a potential conflict of interest.

